# Incidental giant renal schwannoma misdiagnosed as angiomyolipoma and low-grade spindle cell neoplasm: A case report

**DOI:** 10.1016/j.ijscr.2025.112011

**Published:** 2025-10-06

**Authors:** Anahita Ansari Djafari, Sina Samenezhad, Mohammad Seifipoor, Dorna Rafighi

**Affiliations:** a Urology Department, School of Medicine, Shahid Beheshti University of Medical Sciences, Tehran, Iran; b Department of Microbiology, Faculty of Basic Sciences, Research Sciences Branch, Islamic Azad University, Tehran, Iran

**Keywords:** Renal schwannoma, Misdiagnosis, PCNL, Retroperitoneal mass, Core needle biopsy, Spindle cell neoplasm

## Abstract

**Introduction and importance:**

Renal schwannomas are extremely rare benign tumors of the peripheral nerve sheath, accounting for less than 1 % of renal masses. Due to their nonspecific clinical and radiologic features, they are frequently misdiagnosed, posing a diagnostic challenge.

**Case presentation:**

We report a 63-year-old male with chronic right flank pain and a right staghorn calculus. During preoperative evaluation for percutaneous nephrolithotomy (PCNL), a large retroperitoneal mass adjacent to the left kidney was incidentally found. Initial core needle biopsy suggested angiomyolipoma, later revised to a low-grade spindle cell neoplasm. Complete surgical resection via retroperitoneal approach was performed. Histopathology confirmed a diagnosis of schwannoma with typical Antoni A and B areas.

**Clinical discussion:**

Renal schwannomas can mimic various renal neoplasms radiologically and histologically. Core needle biopsy may be misleading in heterogeneous tumors like schwannomas. Complete excision followed by histopathologic evaluation remains essential for definitive diagnosis. A multidisciplinary approach is critical to avoid misdiagnosis and guide optimal management.

**Conclusion:**

This case emphasizes the importance of considering schwannoma in the differential diagnosis of retroperitoneal masses. Surgical resection with thorough pathological analysis is key to accurate diagnosis and effective treatment.

## Highlights

• Rare renal schwannoma misdiagnosed as angiomyolipoma and spindle cell tumor
• Mass discovered incidentally during PCNL workup for staghorn kidney stone
• Accurate diagnosis made only after complete retroperitoneal excision
• Core needle biopsy was misleading due to tumor's heterogeneous nature.
• Highlights need for expert pathology and multidisciplinary team approach.

## 1 Introduction

Schwannomas are exceptional benign tumors arising from peripheral nerve sheath. Schwannomas are almost anywhere in the body including head, neck, limbs and. Renal schwannomas are exceptionally rare, representing less than 1–3 % of all reported cases [1].

Preoperative diagnosis is almost impossible due to impaired characteristic patterns for diagnosing Schwannomas. Thus, these cases are diagnosed usually during pathologic examinations. Most of renal Schwannomas are based in renal parenchyma imitating renal cell carcinoma features, also they can originate from renal hilum because of parasympathetic nerve roots just beside renal artery [2].

In this case we present incidental case of huge Schwannoma during preparation for Percutaneous nephrolithotomy. The patient was treated with retroperitoneal total resection of intact mass. The pathology report stated that the mass was renal schwannoma. This case report has been reported in line with the SCARE checklist [3].

## 2 Case presentation

**Fig. 1:**
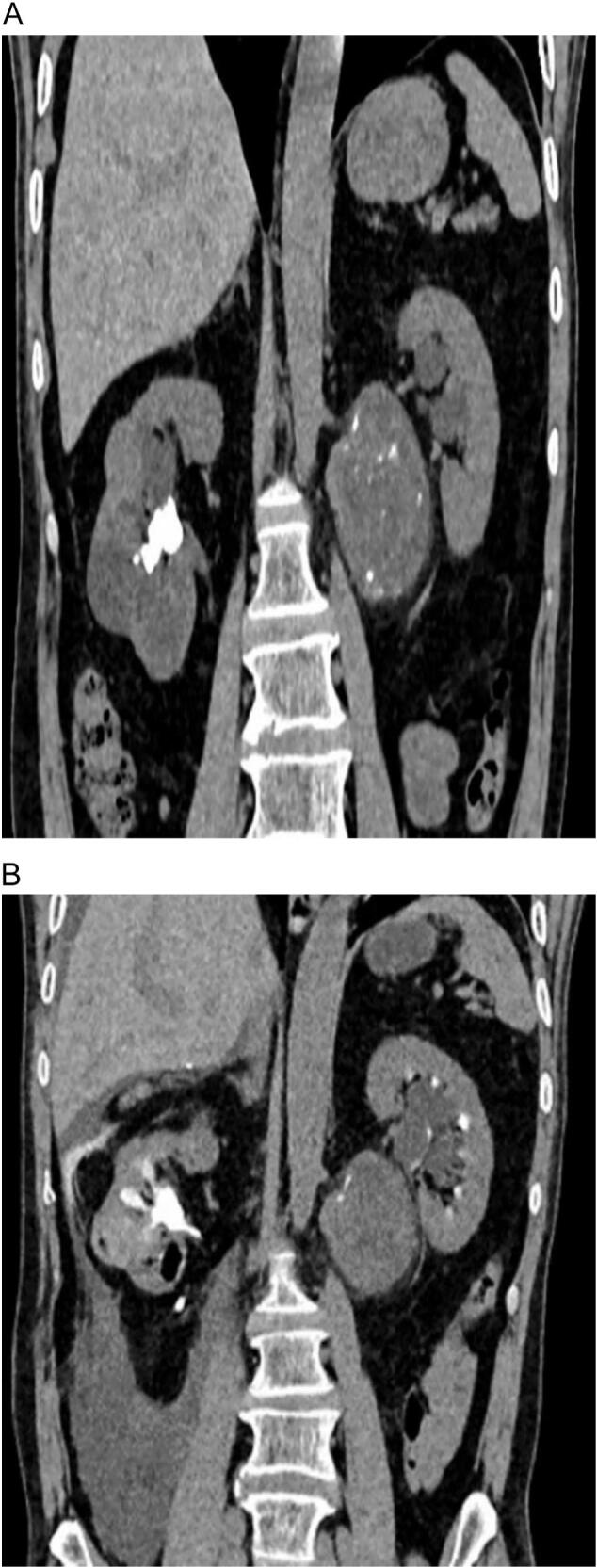
A. CT shows staghorn stone in right kidney and unknown origin mass beside left kidney. B. contrast enhanced CT showed no enhancement.

**Fig. 2:**
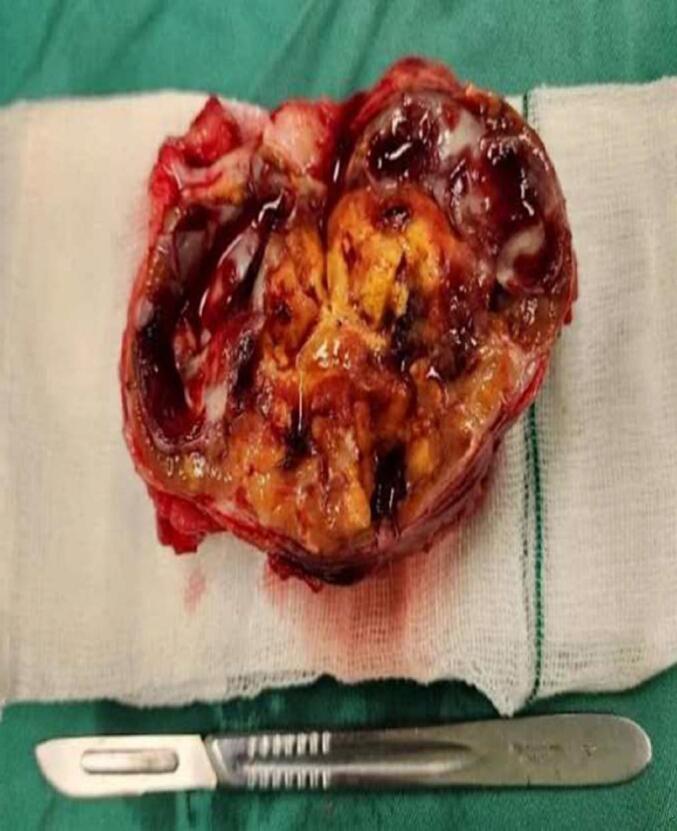
Gross view of the mass mimicking renal cyst or xanthoma.

**Fig. 3:**
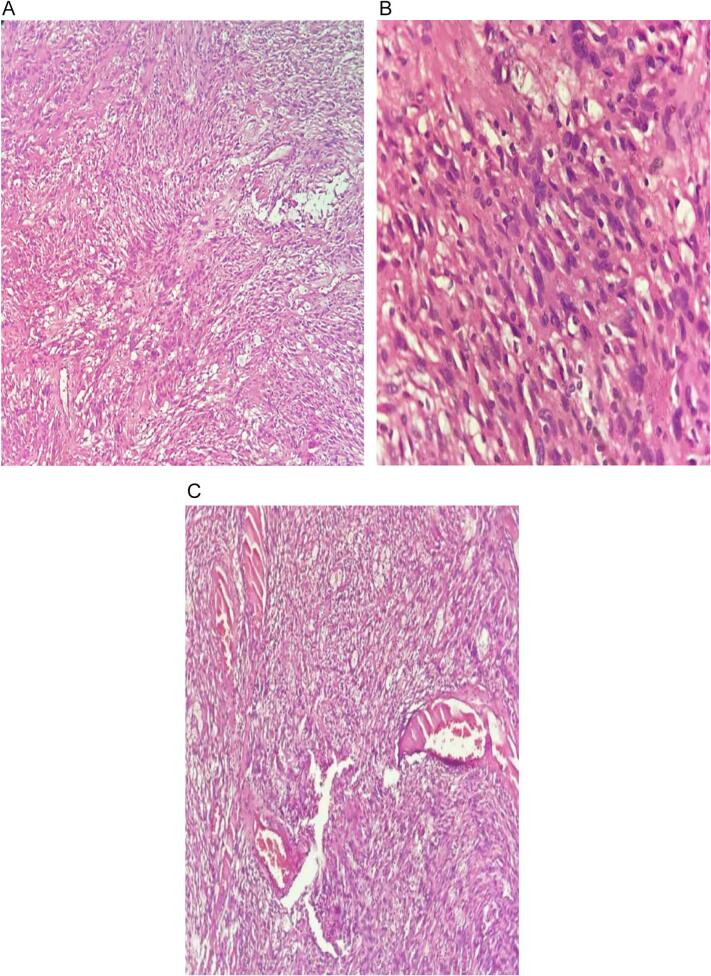
A. Antoni A & Antoni B areas. B. Whorling pattern and oval or wavy nuclei and eosinophilic pattern of Antoni A. C. Hypocellular and spindle cells in loose stroma of Antoni B.

A 63-year-old male patient presented to urology outpatient with chronic right flank pain of a month duration. He had no history of flank pain or urologic symptoms. No history of urological malignancies in family was noted. Physical examination was unremarkable, just a fine costovertebral angle tenderness of right flank without palpable flank mass. Computed tomographic images revealed a 19 ∗ 23 mm staghorn stone with moderate hydronephrosis of right kidney, there is an incidental finding of 68 ∗ 71 ∗ 61 solid cystic and calcified mass beside medial of left kidney and paraaortic region (Fig. 1-A). With inadequate information given by CT, we decided to do contrast enhanced CT to expose the indeterminate mass (Fig. 1-B) demonstrated a heterogeneous, solid-cystic retroperitoneal mass with areas of calcification and poor contrast enhancement. The lesion displaced the left renal hilum and compressed adjacent vessels. Without our knowledge, patient himself in another hospital and with consultation of general surgeon underwent core needle biopsy and diagnosed as angiomyolipoma. We decided to ask second opinion from expert pathologist and pathology report came as low-grade spindle cell neoplasm. in our case, we elected to first address the right kidney stone with PCNL, as the patient presented with chronic right flank pain and hydronephrosis due to a staghorn calculus. Ensuring adequate right renal function was prioritized before undertaking a major retroperitoneal resection on the contralateral side. While DJ stenting of the right kidney could theoretically have been performed, definitive clearance of the stone pathology was considered the safer option to preserve renal function long term. After 4 weeks with patient informed consent complete resection of intact mass with subcostal access was done. In action we found out that the mass was in the Gerota fascia pushing renal hilum and renal artery up. The mass itself had a vascular connection to aorta that we managed to carefully dissect. There were no complications during surgery. Creamy brown elastic tissue measuring85 ∗ 70 ∗ 35 mm yellowish ill-defined mass with hemorrhagic area received by pathology department (Fig. 2). Pathology report stated that the peritoneal mass was Schwannoma. Pathology of Schwannoma is defined by cellular region named as Antoni A, composed of bundles of spindle cell, and hypocellular Antoni B region, composed of randomly arranged spindle cells in myxoid stroma (Fig. 3-A). Antoni A areas with whorling pattern and oval or wavy nuclei and eosinophilic pattern. Antoni B areas are hypocellular and consist of spindle cells in loose stroma (Fig. 3-B & C). The patient has been followed for 12 months postoperatively with no evidence of recurrence or residual disease on imaging and preserved renal function.

## 3 Discussion

The very first report of schwannoma came by verocay in the early 19th century. 30 years later masson found out that nerve sheath is the core. Schwannoma are rare and renal Schwannomas are super rare. we Summarized all cases of renal schwannoma reported in the English literature from 1955 to 2020 with patient age, sex and the location of tumor individually in Table 1 [1,4,[5], [6], [7], [8], [9], [10], [11], [12], [13], [14], [15], [16], [17]].

**Table 1:** Summary of renal schwannoma cases reported in the English literature.

Author/References	Patient age/Gender	Location	Diagnosis
Phillips [3] 1955	56Y/M	Lt pelvis	Schwannoma
Fein [4] 1965	51Y/F	Rt pelvis	Schwannoma
Bair [5] 1978	56Y/M	Rt hilum	Schwannoma
Steers [6] 1985	50Y/F	Rt hilum	Schwannoma
Kitagawa [7] 1990	51Y/M	Lt hilum	
Somers [8] 1988	55Y/F	Lt intraparenchymal	Schwannoma
Hung [9] 2007	36Y/F	Lt intraparenchymal	Schwannoma
El-Bahrawy [10] 2008	55Y/M	Rt intraparenchymal	Cellular schwannoma with a lymphatic cuff feature
Gobbo [11] 2008	35Y/F Lt	renal hilum	Schwannoma
Gobbo [11] 2008	27Y/F	Rt intraparenchymal mild-lower pole	Ancient schwannoma
Gobbo [11] 2008	59Y/F	Lt renal hilum	Schwannoma
Sfoungaristos [12] 2011	55Y/F	Renal hilum	Schwannoma
Yang [13] 2012	40Y/F	Lt renal pelvis	Schwannoma with a lymphatic cuff feature
Verze [14] 2014	59Y/M	Rt intraparenchymal upper pole	Schwannoma
Hanshima [15] 2015	56Y/F	Lt renal hilum	Schwannoma
Suping Hou [16] 2018	62Y/F	Lt renal hilum	Primary cellular schwannoma

Because of the slow and silent nature of Schwannomas Early prognosis is almost impossible and they are often found accidentally in patients' routine check-up or with irrelevant signs and symptoms [2]. Lots of imaging protocols have been utilized to narrow differential diagnosis. Contrast enhanced computed tomography and MRI with all the techniques found no exact or even close to exact characteristic pattern to give us a clue to approach this kind of retroperitoneal mass [18]. The matter of concern in this patient was the metastasis itself as retroperitoneum can host variety of both benign and malignant lesions. Peripheral Nerve sheath is the core of schwannoma. Definite regions of hypercellularity & hypocellularity named Antoni A and Antoni B plus company of S100 positivity can confirm the diagnosis of Schwannoma [19]. Ancient Schwannoma is a unique variant of schwannoma with gross degenerative changes like calcification, necrosis, hemorrhagic pattern and hyalinized features imitative of renal cyst, xanthoma and a malignant tumor [20] (see Fig. 3).

As described above, due to variation of components in ancient schwannoma core needle biopsies are quite misleading and complete resection of mass with free margin and pathological evaluation of mass with possibilities of finding malignancies it seems to be the right choice of management [19].

Almost 12 % of schwannomas tend to recur after complete resection with free margin, higher rates of recurrence reported in partial resection and enucleation [21].

The initial core needle biopsy was misleading, reporting angiomyolipoma and later spindle cell neoplasm. This reflects the heterogeneous architecture of schwannomas, where sampling from hypocellular Antoni B areas may resemble other spindle cell tumors. Sampling error and interpretative challenges are well recognized pitfalls, underscoring the importance of complete excision for accurate diagnosis.

Despite all the speculations and all the possible differential diagnosis from a simple renal cyst to renal cell carcinoma and even pathological report of core needle biopsy as angiomyolipoma and spindle cell tumor, the exact diagnosis of mass was Schwannoma confirmed by three different pathologists. We as surgeons must be aware of certain possibilities to ensure patients of getting the best management. The role of imaging and expert radiologists to report and role of biopsies and also tissue painting and even genetic testing should be highlighted before doing surgery.

There are no standardized guidelines for follow-up imaging of renal schwannomas due to their rarity. However, given the risk of recurrence after incomplete resection (reported up to 12 %), we adopted a pragmatic protocol of CT surveillance every 6 months during the first two years, consistent with follow-up strategies for other retroperitoneal tumors.

## 4 Conclusion

Renal schwannomas are exceedingly rare and often pose a diagnostic challenge due to their non-specific clinical and radiologic features. This case highlights the importance of considering schwannoma in the differential diagnosis of retroperitoneal masses, especially when initial biopsy results are inconclusive or misleading. Complete surgical excision remains the gold standard for diagnosis and treatment. A multidisciplinary approach involving radiologists, pathologists, and urologists is crucial for accurate diagnosis and optimal patient management. Long-term imaging follow-up is recommended to monitor for potential recurrence.

## Author contribution

Dr Sina Samenezhad: study concept, data analysis, interpretation, writing the paper

Dr Anahita Ansari Djafari: study concept, interpretation

Dr Mohammad Seifipoorv: data collection

Dr Dorna Rafighi; language editing

## Consent

Written informed consent was obtained from the patient for publication and any accompanying images. A copy of the written consent is available for review by the Editor-in-Chief of this journal on request.

## Ethical approval

This study was reviewed by our hospital Institutional Review Board (IRB) and was deemed exempt from formal ethical approval because the treatment and data collection were based entirely on established clinical guidelines and standard urological practice performed by an expert urologist. No experimental interventions or novel protocols were involved, and all actions followed evidence-based, routine medical care.

## Guarantor

Dr Sina Samenezhad.

## Research registration number

Not applicable.

## Funding

No funding was received for conducting this study.

## Conflict of interest statement

The authors declare they have no financial interests. The authors report no conflict of interest.
